# Thoracic injuries in elderly trauma patients epidemiology, injury characteristics, in-hospital care and outcome

**DOI:** 10.1186/s13049-026-01676-4

**Published:** 2026-08-17

**Authors:** Andrea Lundin, Katarina E. Göransson, Anders Enocson

**Affiliations:** 1 Department of Trauma, Acute Surgery and Orthopaedics, Karolinska University Hospital, Stockholm, 171 64 Sweden; 2 Department of Medicine, Karolinska Institutet, Solna, Sweden; 3 School of Health and Welfare, Dalarna University, Falun, Sweden; 4 Department of Molecular Medicine and Surgery, Karolinska Institutet, Stockholm, Sweden

**Keywords:** Trauma, Chest injury, Thoracic trauma, Poly trauma, Mortality

## Abstract

**Purpose:**

The aim was to describe epidemiology, injury characteristics, in-hospital care and outcome for elderly trauma patients with thoracic injuries within the context of a large national register study.

**Methods:**

Trauma patients aged ≥ 60 years from 2022 to 2024 were identified through the national Swedish Trauma Registry (SweTrau). The Abbreviated Injury Scale (AIS) was used to identify thoracic and other injuries. Data on patient and injury demographics, in-hospital care and outcome in terms of discharge destination and mortality was collected for each patient.

**Results:**

A total of 11,624 patients were included. The median age for all patients was 76 years (range 60–106), and 4617 (40%) were females. A total of 3644 (31%) patients had a thoracic injury registered (TI group). Compared with patients without thoracic injury (NTI), the TI group had higher injury severity (ISS and NISS) and were more physiologically compromised on arrival (lower systolic blood pressure and a higher proportion of patients presenting with circulatory shock (SBP < 90 mmHg). TI patients had more injuries, longer hospital length of stay, required more intensive care and were less often discharged to their home, while 30day mortality did not differ between groups.

**Conclusion:**

Elderly trauma patients with thoracic injuries were more severely injured, required more in-hospital care and could less often be discharged directly to their home, compared to patients without thoracic injury.

## Introduction

Trauma remains a leading contributor to morbidity and mortality worldwide, particularly among individuals under 45 years of age [1]. Head injuries are the predominant cause of death after trauma, followed by abdominal and thoracic injuries [2, 3]. Traumatic thoracic injuries include damage to intrathoracic organs (lungs, heart, vessels, and other organs), ribs and sternum fractures, pulmonary contusions or lacerations, and pneumo or hemothorax [4]. These injuries are associated with a high risk of both primary and secondary complications such as respiratory failure and/or pneumonia [5, 6]. Thoracic trauma has been linked to extended hospitalization and an increased need for intensive care, predominantly due to respiratory complications that necessitate advanced ventilatory support [6, 7]. Beyond respiratory sequelae, thoracic trauma is a major contributor to early traumatic mortality owing to its strong association with noncompressible intrathoracic haemorrhage. Recent evidence identifies the thorax as the most frequent site of fatal haemorrhage among patients who die in hospital from traumatic bleeding [8]. While younger patients with thoracic injuries may face notable morbidity, geriatric patients are particularly vulnerable; they endure a higher incidence of rib fractures and are more likely to develop respiratory failure and pneumonia, resulting in longer hospital stays compared to younger individuals [9]. Hospitalization length increases significantly with the number of rib fractures [10–12]. Additionally, rib fractures are associated with compromised respiratory function and an increased risk of infectious complications, which further complicates clinical management [6, 10, 12–14].

The global population aged 60 and above is expected to double by 2050 [15]. In Sweden, the demographic shift is evident, with individuals aged 65 and older is expected to rise from 20% today to over 25% already within the next two decades [16]. Longer life expectancy in combination with a more active lifestyle contributes to a higher incidence of trauma among elderly individuals. At the same time, age-related physiological changes might increase the risk for complications and mortality associated with trauma [17]. Elderly trauma patients often present with multiple comorbidities, reduced physiological reserve, and altered pharmacokinetics, all of which complicate both acute management and recovery. These factors lead to prolonged rehabilitation needs and an increased demand for tailored care approaches [15].

Despite the recognized clinical complexities associated with thoracic trauma in elderly adults, there is a lack of trauma-specific management guidelines tailored to this patient group. Consequently, understanding the epidemiology and injury characteristics within this patient group is critical. This knowledge is essential for improving clinical care and decision-making, as well as for guiding future research aimed at enhancing outcomes for elderly trauma patients with thoracic injuries. This study aimed to describe epidemiology, injury characteristics, injury severity, in-hospital care, and outcome for elderly trauma patients with thoracic injuries within the context of a large national register study.

## Patients and methods

### Study population

The Swedish Trauma Registry (SweTrau) is a national quality registry for trauma care in Sweden established in 2011. The registry is serving to improve trauma care by analysing trauma characteristics, treatment outcomes, and quality indicators related to the management of trauma patients. In recent years, 94% of all emergency hospitals that provide 24-hour emergency care report data to SweTrau [18]. Variables in the SweTrau are registered according to the Utstein template to enable international comparisons with other trauma centers [19]. SweTrau includes patients who arrive with trauma activation, or who arrive without a trauma alert but have a New Injury Severity Score (NISS)>15. The registry has been reported to have high accuracy, correctness, and completeness [20].

The study period was January 1^st^, 2022, to December 31^st^, 2024, and all patients aged 60 years or older at the time of injury who were admitted to in-patient care units in Sweden and registered in SweTrau were identified. Patients without any injuries were excluded, as well as patients with inadequate age registration. Patients sustaining thoracic injuries were identified using the Abbreviated Injury Scale (AIS), a coding system for injured body regions and injury types. Thoracic injuries were further sub-classified according to the AIS codes. Data gathered for each patient included: patient characteristics (age, gender and ASA-class), injury characteristics (number and types of injuries), injury severity (ISS (Injury Severity Score), NISS, GCS (Glasgow Coma Scale) and vital signs. In addition, data on in-hospital care (length of stay and intensive care), discharge destination and mortality was collected.

### Statistical methods

Categorical data were displayed using frequency counts and percentage distributions. The variables were analyzed using Fisher’s exact test. Numerical data were presented as medians with interquartile ranges (IQRs), unless otherwise stated. Comparisons between independent groups were conducted using the Mann–Whitney U test. All statistical tests were two-tailed. The results were considered statistically significant at *p* < 0.05. Multivariable logistic regression was used to analyse thoracic injury (yes or no), 30-day mortality (yes or no), hospital length of stay (0–4 or >4 days, 4 days = median for all patients) and ICU-care (yes or no) with stepwise addition of confounders; gender, ASA-class (1–2 or 3–4), NISS (<15 or ≥15), head injury (yes or no). Results were presented as odds ratios (ORs) with 95% confidence intervals (95% CIs). The statistical software used was IBM SPSS Statistics, version 29 for Windows (SPSS Inc., Chicago, Illinois).

## Results

### Epidemiology and patient characteristics

**Fig. 1:**
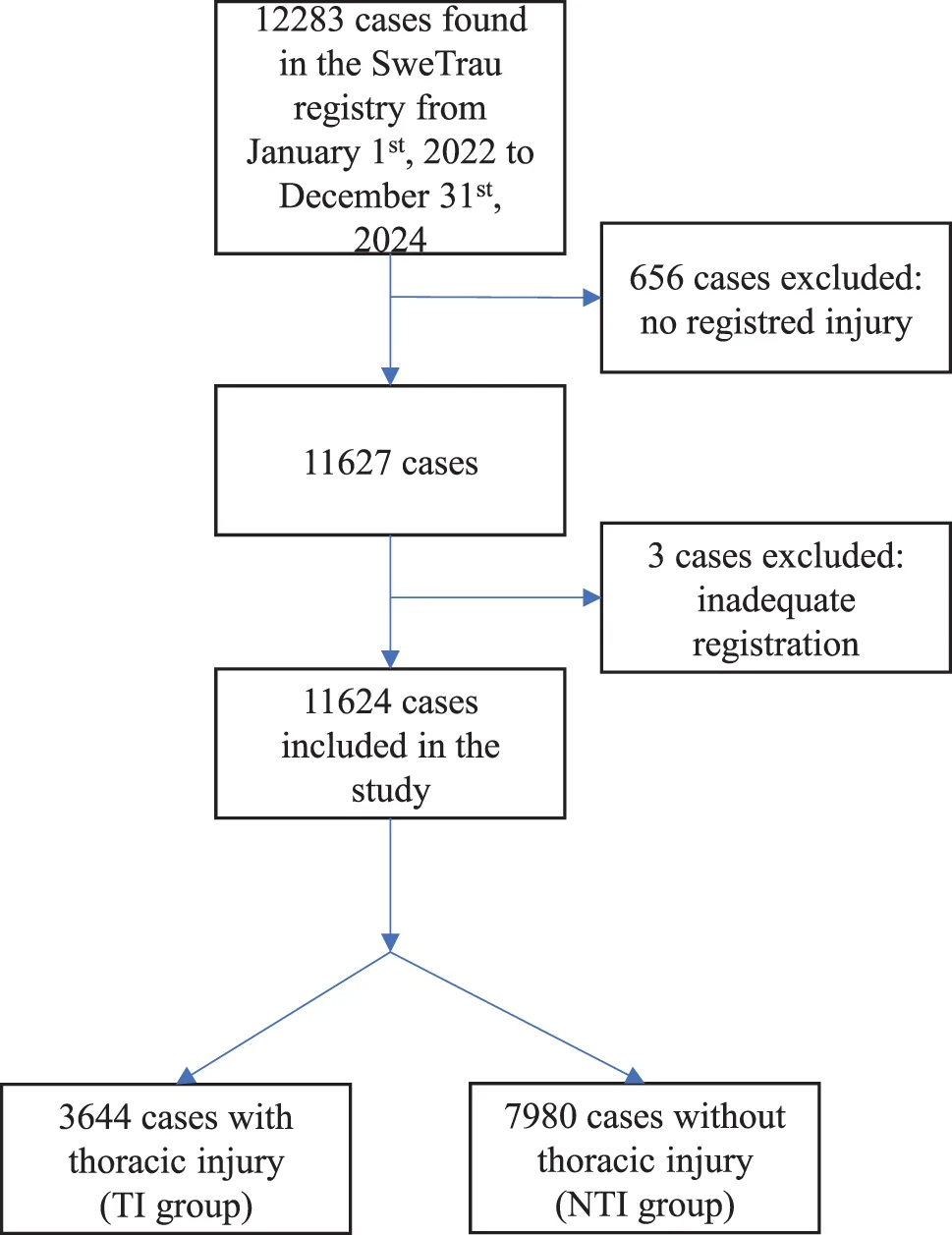
Flowchart of the inclusion of participants in the study

Initially, 12,283 cases were found in the SweTrau registry from January 1^st^, 2022, to December 31st, 2024. After exclusion of 656 cases without a registered injury and 3 cases due to inadequate age registration (>120 years), a total of 11,624 patients were included in the analyses (Fig. 1). The median age for all patients was 76 years (range 60–106), and 4617 (40%) were females. A total of 3644 (31%) had a thoracic injury registered (TI group). There was a higher proportion of males in the TI group than in the non-thoracic injury (NTI) group (67%, *n* = 2427 versus 57%, *n* = 4580, *p* < 0.001). There was a lower proportion of ASA-class 3–4 in the TI group (46%, *n* = 1657) compared to the NTI group (53%, *n* = 4187) (*p* < 0.001) (Table 1). The most common injury region for all patients was the head (47%, *n* = 5408), followed by upper limbs (33%, *n* = 3794) and the chest (31%, *n* = 3644). Detailed data on injury mechanisms and injury regions are presented in Table 1.

**Table 1:** Epidemiology, injury characteristics, vital signs, ICU care, hospital length of stay and mortality

Variable	All patients (n = 11624)	Patients with thoracic injury (n = 3644)	Patients without thoracic injury (n = 7980)
**Age; Median (IQR)**	76 (67–84)	75 (67–82)	76 (68–84)
**Gender Female; n= (%)**	4617 (40)	1217 (33)	3400 (43)
**ASA-class 3–4; n = (%)**	5844 (50)	1657 (46)	4187 (53)
**Injury mechanism; n= (%)**			
High fall Low fall MVA MC Bicycle Gunshot Stabbing Pedestrian Explosion Other vehicle Blunt force Other	2799 (24) 5161 (44) 1150 (10) 355 (3.1) 790 (6.8) 26 (0.2) 260 (2.3) 306 (2.7) 15 (0.1) 96 (0.8) 383 (3.3) 195 (1.7)	1047 (29) 1026 (28) 638 (18) 210 (5.8) 279 (7.7) 3 (0.1) 47 (1.3) 110 (3.0) 1 (<0.1) 41 (1.1) 157 (4.3) 73 (2.0)	1752 (22) 4135 (52) 512 (6.5) 145 (1.8) 511 (6.5) 23 (0.3) 213 (2.7) 196 (2.5) 14 (0.2) 55 (0.7) 226 (2.9) 122 (1.5)
**Type of injury; n= (%)**			
Blunt Penetrating	11287 (97) 315 (2.7)	3582 (99) 56 (1.5)	7705 (97) 259 (3.3)
**ISS; Median (IQR)**	9 (4–14)	13 (8–18)	6 (2–13)
**ISS > 15; n= (%)**	2873 (25)	1321 (36)	1552 (19)
**NISS; Median (IQR)**	11 (4–19)	17 (19–22)	9 (3–17)
**NISS > 15; n= (%)**	4380 (38)	2021 (56)	2359 (30)
**GCS; Median (IQR)**	15 (14–15)	15 (15–15)	15 (14–15)
**GCS < 9; n= (%)**	982 (8.7)	335 (9.5)	647 (8.4)
**SBP (mmHg); Median (IQR)**	142 (125–160)	140 (120–158)	145 (127–161)
**Shock; n= (%)**	338 (3.1)	136 (4.0)	202 (2.7)
**Head injury; n= (%)**	5408 (47)	1230 (34)	4178 (52)
**Face injury; n= (%)**	3426 (30)	892 (25)	2534 (32)
**Neck injury; n= (%)**	488 (4.2)	143 (3.9)	345 (4.3)
**Abdominal injury; n= (%)**	900 (7.7)	510 (14)	390 (4.9)
**Spine injury; n= (%)**	2784 (24)	1174 (32)	1610 (20)
**Upper limb injury; n= (%)**	3794 (33)	1475 (41)	2319 (29)
**Lower limb injury; n= (%)**	3445 (30)	1202 (33)	2243 (28)
**Hospital length of stay (days); Median (IQR)**	4 (2–8)	5 (2–10)	3 (2–7)
**ICU-admissions n= (%)**	2415 (21)	1023 (28)	1392 (17)
**Mortality 30 days; n= (%)**	1478 (13)	481 (13)	997(13)

IQR = Interquartile Range, ASA = American Society of Anesthesiologists, MVA = Motor Vehicle Accident, MC = Motorcycle, ISS = Injury Severity Score, NISS = New Injury Severity Score, GCS = Glasgow Coma Scale, SBP = Systolic Blood Pressure, Shock = SBP<90 mmHg, ICU = Intensive Care Unit

### Injury severity and characteristics

Patients in the TI group had a higher median ISS (13, IQR 8–18) than those in the NTI group (6, IQR 2–13) (*p* < 0.001). The same was for the proportion of patients with ISS > 15 (36%, *n* = 1321 versus 19%, *n* = 1552, *p* < 0.001). A similar pattern was observed for NISS, with higher median scores in the TI group (17, IQR 9–22) compared to the NTI group (9, IQR 3–17) (*p* < 0.001), and a greater proportion with NISS > 15 (56%, *n* = 2021 versus 30%, *n* = 2359, *p* < 0.001). Physiologically, TI patients also arrived at hospital in a more compromised state, with a lower median systolic blood pressure (SBP) (140, IQR 120–158 versus 145, IQR 127–161 mmHg, *p* < 0.001), and a higher the proportion of patients presenting with circulatory shock (SBP < 90 mmHg) in the TI group (4.0%, *n* = 136), versus to the NTI group (2.7%, *n* = 202) (*p* < 0.001). There was no difference in the proportion of patients with GCS < 9 on arrival between patients (TI group 9.5%, *n* = 335 versus NTI group 8.4%, *n* = 647, *p* = 0.06). The median total number of injuries was higher in the TI group (5, IQR 3–8) than in the NTI group (3, IQR 2–4) (*p* < 0.001). The most common injury mechanism in both groups was a low fall (44%, *n* = 5161), followed by high falls (24%, *n* = 2799) and motor vehicle accidents (10%, *n* = 1150). Only 2.7% of the injuries were caused by a penetrating trauma.

### Thoracic injuries

Among the 3644 patients with thoracic injury, the most common injury was a rib fracture (76%, *n* = 2775), and 2081 (57%) had ≥ 3 rib fractures (Table 2). The second most common thoracic injury was a pneumothorax (29%, *n* = 1047), followed by a haemothorax (18%, *n* = 660). There were some gender differences in the distribution of thoracic injuries. Rib fractures, including multiple rib fractures, and haemothorax occurred more frequently in males. No significant differences were found in the gender distribution for sternum fractures, pneumothorax, lung contusion, vascular injuries or heart injuries. Detailed data on thoracic injuries, including relation to gender, are presented in Table 2.

**Table 2:** Type of thoracic injury in relation to gender

Injury type	All patients with thoracic injury (n = 3644) *	Gender Female (n = 1217) Male (n = 2427) p-value		
Rib facture; *n*= (%)	2275 (76)	891 (73)	1884 (88)	0.03
Multiple rib fracture (≥3)	2081 (57)	31 (54)	1427 (59)	0.004
Pneumothorax; *n* = (%)	1047 (29)	290 (24)	757 (32)	<0.001
Hemothorax; *n*= (%)	660 (18)	167 (14)	493 (20)	<0.001
Sternum fracture; *n*= (%)	459 (13)	160 (13)	299 (12)	0.5
Lung contusion; *n*= (%)	418 (12)	125 (10)	293 (12)	0.1
Vascular injury; *n*= (%)	44 (1.2)	15 (1.2)	29 (1.2)	1.0
Heart injury; *n*= (%)	38 (1.0)	14 (1.2)	24 (1.0)	0.7

### Hospital length of stay, ICU care, discharge destination and mortality

The median hospital length of stay was longer in the TI group (5, IQR 2–10 days) compared to the NTI group (3, IQR 2–7 days) (*p* < 0.001). This was confirmed (OR 1.5, 95% CI 1.2–1-5, *p* < 0.001) with multivariable logistic regression analysis adjusting for gender, ASA-class, NISS and head injury. The proportion of patients admitted to intensive care unit (ICU) was higher in the TI group (28%, *n* = 1023) than in the NTI group (17%, *n* = 1392) (*p* < 0.001). This difference persisted (OR 1.2, 95% CI 1.1–1-3, *p* = 0.003) with multivariable logistic regression analysis adjusting for gender, ASA-class, NISS and head injury. Of those patients who were alive at discharge (*n* = 10563), there was a lower proportion of patients who were discharged directly to their home in the TI group (57%, *n* = 1855) compared to the NTI group (61%, *n* = 4460) (*p* < 0.001). There was no difference in 30-day mortality between groups (TI group: 13%, *n* = 481, versus NTI group: 13%, *n* = 997, *p* = 0.3). No increased 30-day mortality (OR 0.9, 95% CI 0.8–1.1, *p* = 0.4) could be identified for patients with thoracic injury with multivariable logistic regression analysis adjusting for gender, ASA-class, NISS and head injury.

### Missing data

Missing values were most common for SBP (*n* = 813), followed by GCS (*n* = 401). Additional missing data included injury mechanism (*n* = 88), ASA classification (*n* = 46), type of injury (*n* = 22), discharge destination (*n* = 15), hospital length of stay (*n* = 5) and ICU stay (*n* = 3). All statistical analyses were performed using available data only.

## Discussion

We found that geriatric trauma patients with thoracic injuries (TI) presented with substantially greater injury severity and required more intensive hospital resources compared with geriatric trauma patients without thoracic injuries (NTI). Patients in the TI group had significantly higher ISS and NISS scores, arrived in a more physiologically compromised state, and sustained a greater total number of injuries. These findings are consistent with previous research demonstrating that thoracic injuries, particularly rib fractures, pneumothorax, and haemothorax, are strongly associated with increased injury burden and physiological derangement in elderly patients [21, 22]. When we analysed different types of thoracic injuries, we found correlations between gender and some specific injuries. However, as no adjustments were made for confounders, we think that these results should be interpreted with caution.

Findings from a large national study in the United States on blunt thoracic trauma indicated that elderly patients with severe blunt thoracic injuries were more likely to require ICU care [23]. This is in line with our study, which showed that a higher proportion of TI patients require intensive care. We interpret this as these injuries are a major driver of critical care needs, especially in elderly with reduced physiological reserve. Regarding the pattern of thoracic injuries, rib fractures represented the most frequently observed injury (76%). Similar findings have been reported by Sirmali et al. [24], who found that rib fractures were present in 47% of their cases. However, the cohort in their study was considerably smaller, comprising only 261 cases.

The longer hospital length of stay observed among TI patients in our cohort may be explained by prior reported findings, indicating that patients with thoracic injuries require prolonged pain management, respiratory support, and close monitoring to prevent deterioration [25, 26]. This greater resource utilisation observed in the TI group is in line with health economic studies showing that thoracic injuries, particularly multiple rib fractures, substantially increase healthcare costs due to longer hospital stays, greater ICU use, and increased need for rehabilitation services [25, 27]. Given the ageing population and the predominance of lowenergy falls as a mechanism of injury (accounting for 44% of cases in our cohort) this represents a growing challenge for trauma systems. This pattern is further supported by a nationwide Dutch study by Peek et al. [28], which similarly reported that 43% of elderly trauma patients sustained their injuries through a low-energy mechanism, thereby reinforcing the increasing clinical and organisational challenges associated with such injuries in an ageing society.

Although our study did not include complication data, the increased ICU utilisation and longer hospital stay observed in the TI group likely reflect the clinical vigilance required to prevent adverse events such as respiratory complications. Our results highlight that early and proactive management strategies including aggressive pain control, respiratory physiotherapy, and close monitoring to mitigate the risk of deterioration, are of utmost importance [27, 29].

Although research on the full spectrum of thoracic injuries in elderly adults remains limited, several studies have examined specific injury types, such as rib fractures [30], pneumothorax, and haemothorax [31]. While a recent Turkish study by Çetin et al. [32] provided valuable insights into thoracic trauma among elderly adults, its findings are limited by the relatively small cohort of 261 patients from a single centre.

Our findings indicate that patients in the TI group presented with poorer overall condition, sustained more severe injuries, and required greater healthcare resources, including increased use of intensive care and longer lengths of stay on inpatient wards. Therefore, most of these patients could not be discharged home, but instead required continued inpatient rehabilitation or followup care. Given the vulnerability of this patient group, effective discharge planning becomes a central component of their recovery, ensuring continuity of care [33]. By examining the entire continuum of care (including discharge destination), our study highlights different phases in which these patients require additional support to achieve more favourable outcomes. The absence of any frailty measure in the present analysis warrants further consideration, as relying solely on chronological age and ASA-class may inadequately capture the heterogeneity of elderly trauma patients. Contemporary evidence consistently shows that frailty is a stronger predictor of adverse outcomes - including mortality, complications, and discharge disposition - than age alone [34, 35]. In this context, it is also relevant that the current national trauma registry does not yet include frailty variables, although there is an ongoing initiative to implement such measures in the coming years, which may substantially improve both clinical risk stratification and future research. We found no difference in 30day mortality between TI and NTI patients. However, long-term mortality differences may still emerge, as elderly trauma patients often experience delayed functional decline and increased long-term mortality [36, 37]. Future studies incorporating longterm followup are therefore warranted. One can argue about our decision of a 60-year age cut-off, however it is a commonly applied threshold in trauma research, frequently used in studies examining outcomes and survival [38–40].

Overall, our findings emphasise that thoracic injuries in elderly adults represent a highrisk clinical entity associated with greater injury severity, increased need for intensive care, longer hospital stays, and substantial healthcare resource utilisation. These results reinforce the importance of early identification, structured management pathways, and heightened clinical vigilance to prevent complications and optimise outcomes in this vulnerable patient population.

### Strengths and limitations

The major strength of this study was the use of a national trauma registry with a large and unselected patient population, providing broad representation across trauma mechanisms and hospital settings. However, as the data originates from a registry, the analyses were retrospective and the results descriptive, and must therefore be interpreted with some caution. Another strength was that the use of the Swedish national identification number ensured that only foreigners were lost to follow-up for mortality. A limitation was that data entry into the registry is performed manually, introducing the risk of missing or incorrectly recorded variables. In addition, the nature of the register data (de-identified) further limits the ability to control for all potential confounders, and variations in documentation practices across hospitals may affect data quality. Another limitation was that the national registry does not capture data on complications and adverse events, such as respiratory complications, which would have been highly relevant for this study. Finally, although this was a large patient series, it was still a national cohort and therefore might not be representative of all other countries and regions.

## Conclusion

Elderly trauma patients with thoracic injuries were more severely injured, had more injuries, longer hospital length of stay, were more often admitted to intensive care, and could less often be discharged directly to their home, compared to patients without thoracic injury.

## Data Availability

No datasets were generated or analysed during the current study.
